# Intensified discrimination against tenants and its health effects during the COVID-19 pandemic in large Chinese cities

**DOI:** 10.1038/s41598-023-48935-3

**Published:** 2023-12-15

**Authors:** Lu Shan, Shenjing He

**Affiliations:** 1 Department of Urban Planning and Design, Urban Systems Institute, and the Social Infrastructure for Equity and Wellbeing Lab, The University of Hong Kong, Hong Kong Special Administrative Region, China; 2 Department of Urban Planning and Design, Urban Systems Institute, and the Social Infrastructure for Equity and Wellbeing Lab, The University of Hong Kong, Room 836A, 8/F, Knowles Building, Pokfulam Road, Hong Kong, China

**Keywords:** Environmental social sciences, Risk factors

## Abstract

The COVID-19 pandemic in many senses reconstructs social norms and reshapes social behaviour, which typically assumes a close correlation between mobility with a higher risk of COVID-19 infection. This may intensify the pre-existing discrimination against tenants and widen tenure-based health inequalities. Drawing on an online questionnaire survey conducted in five major cities in China in 2020, we employ multi-level regression models to examine the intensified discrimination against tenants during COVID-19 and its impacts on residents’ physical and mental health inequalities. Results show that the pre-existing inequalities have been intensified during COVID-19 and the perceived discrimination has rendered worsened self-rated health and mental health and enlarged health inequalities. The discrimination particularly affected tenants with better economic profiles or worse health conditions; by contrast, despite being exposed to more tenant-related discriminatory experiences, rural *hukou* holders suffered from less severe health inequalities. A clear linkage is found between renting in poorly-managed and larger health gaps generated by discrimination. The negative health impact of intensified discrimination is found to be more significant in communities with lower infection risk, which points to the necessity of understanding the long-term health impact of discrimination against tenants in a more holistic way. In terms of community environment, we discover a positive effect of community social capital, i.e., higher level social capital helps mitigate the health threat of discrimination against tenants during COVID-19. Besides, public housing tenants reported better health outcomes and were less exposed to intensified discrimination during COVID-19 than private housing tenants. These findings provide a nuanced understanding of variations determined by individual and territorial factors, thus present timely policy implications for promoting healthy and inclusive urban development in the post-pandemic era.

## Introduction

In the contemporary society where social groups are exposed to an increasingly unpredictable environment, housing is described as ‘one of the major environmental, as well as social determinants of public health’^1^. In the housing literature exploring the health impact of housing attributes, the link between housing tenure and health is one of the most common and important issues^2^. Housing tenure is typically categorized into four types: homeowner without a mortgage, homeowner with a mortgage, tenant renting in private housing, and tenant renting in public housing^2,3^. Health geographers and policymakers are particularly interested in whether and how rental housing in particular is associated with poorer physical and mental health beyond individual socio-economic profiles^4^. For instance, Bloze and Skak observe a higher level of psychological distress for renters than homeowners in Denmark^5^. Generally, the health consequences of housing tenure are typically documented as lower rates of heart disease, lower age-adjusted mortality, better mental health and self-rated health for homeowners compared with renters^6–8^.

What is yet to be fully explored is the discrimination against tenants and how it impacts the health disparities between homeowners and renters. In addition to the disadvantageous socioeconomic status of renters, tenure-based discrimination is another important disadvantage for tenants in contemporary urban communities^9^. Previous studies show that the negative attitudes toward tenants are largely shaped by the social, economic and cultural stereotypes associated with tenants^10^. It is widely acknowledged that the cumulative effect of perceived discrimination as a stressor is a well-received approach to understanding the impact of discrimination on long-term health outcomes^11^. The negative effects of discrimination on physical health may also be partly associated with increased psychological distress^12^. Notably, extant studies also show that these negative health effects of discrimination could be enhanced or weakened by various physical or social community features, such as housing characteristics^13,14^ and social capital within the community^15^. Studies on perceived discrimination suggest an indirect implication of housing tenure leading to health inequalities, although the empirical evidence remains insufficient.

The COVID-19 pandemic has exposed the deep-seated social and political fissures within cities and communities, and further demonstrated the fundamental role of secured and affordable housing in health and well-being. Governments announced a bundle of pandemic control policies to regulate people’s travel patterns and living habits, through which the ‘home front’ has become the ‘new front line’ of public health measures^16^. The extremely high transmission of COVID-19 variants magnifies the health risk associated with frequent mobility, thus leading to the stigmatization of tenants as ‘increasing infection risk in communities^17^. Consequently, the pre-existing discrimination against tenants was intensified^18^. Extensive research has shown that individuals being considered associated with a higher infection risk during epidemic/pandemic outbreaks often face personal stigmatization, resulting in significant negative impacts on their health^19,20^. Most of these studies focus on the stigmatization of particular groups with high infection risks (such as AIDS^21^ and SARS^22^), but broader patterns of marginalization and exclusion experienced by wider social groups have not been sufficiently explored. Existing reports suggest that^23–26^, given the increasingly strengthened control measures and growing inconveniences in daily life during the three years’ pandemic, more tenants in urban areas may be exposed to severe discrimination due to the intensified stigma. However, few studies have examined the indirect implication of COVID-19 on human health, particularly the intensified discrimination against tenants and its health risks.

In recent years, there has been a growing body of literature in China that examines the relationship between housing tenure and health and well-being outcomes in urban areas. The market-oriented housing reform in China has led to soaring property prices that are unaffordable for many families^27^, therefore homeowner identity is usually associated with high socio-economic status^28,29^. Moreover, the long-standing household registration system in urban China has established a direct connection between homeownership and citizenship rights in cities, granting homeowners broader access to urban resources and public services^30,31^. Under these circumstances, housing has de facto become an institution for exclusion and marginalization, which creates a stereotypical tenant identity associated with lower socio-economic status and increased health risks^31^. Renters in urban areas, particularly rural migrants, often encounter discrimination and marginalization, while homeownership in urban China has been found to have a significant and positive impact on individuals' perceived happiness^32^ and life satisfaction^33^. However, empirical studies on this topic remain insufficient.

During the COVID-19 pandemic in China, the central government implemented a comprehensive virus containment strategy known as 'dynamic zero.' This strategy involves various measures such as inter-city commuter registration, inner-city blockades of districts with confirmed cases, and community-level daily nucleic acid testing of residents. These lockdown regulations in Chinese cities are commonly stricter than those in rural areas, partly owing to the higher risks in the urban areas associated with higher population density and mobility. While these regulations aim to control the spread of the virus, they also have the unintended consequence of potentially exacerbating social exclusion against tenants in China. For instance, during the pandemic, residents appear to dislike and even stigmatize those subdivided rental units with extremely high density, owing to the potential high infection risks as reported on social media^34^. Moreover, some media reports suggest that renters are more likely to violate the governance regulations and move outside the pandemic-segregated housing, while rental housing and migrants are always highlighted in pandemic regulations issued during the pandemic^35,36^. Therefore, it is crucial to investigate how the inconvenience caused by strict control policies, as well as the fear of infection risk, may exacerbate social exclusion against tenants in China. This is particularly relevant given the complex dynamics of the pandemic and the potential for discriminatory experiences to impact health outcomes.

In this study, we aim to examine the impact of the COVID-19 pandemic on discrimination against tenants and its implications for health inequalities in urban China. Specifically, based on a dataset of 4880 online questionnaires collected in five major cities in 2020, we are interested in whether the pandemic has intensified discrimination against tenants and to what extent it affects health disparities based on housing tenure. We propose two major hypotheses for this study. Firstly, we hypothesize that the intensified discrimination against tenants during the COVID-19 pandemic will have significant negative health effects, which may vary based on population characteristics. Secondly, we posit that the negative health effects resulting from intensified discrimination can be mitigated to some extent by social capital within communities. The remainder of this paper is organised as follows: we first introduce the methodologies, and then explain the analysis results, conclusions, and limitations.

## Data and methodology

### Study area and data collection

Given the geographical pattern of the confirmed cases in May 2020 in China, we select residents from the eastern, central and western regions: Ha’erbin, Beijing, Shanghai, Wuhan and Chongqing, to include the varieties of regional socio-economic characteristics. By entrusting a professional institution (NetEase), we conduct the questionnaire survey on residents over 18 years old in selected communities. Over 95% of the respondents are from urban areas. All the participants need to accept online informed consent before answering the questionnaire.

This study generates a unique code for each qualified questionnaire according to the city name, community name and statistician code. Trap questions are used to screen participants who do not answer carefully, and the exclusion criteria include (1) the consistency of the questionnaire in the same community is unreasonable (e.g. the description of community location is inconsistent), (2) respondents completed the questionnaire in less than 10 min (the shortest time in the test) and (3) invalid questionnaire code.

### Variable measurement

Based on the survey data, two groups of variables related to health and discrimination in COVID-19 are analysed. For the health outcomes, SRH and mental health are included to evaluate respondents’ health disparities in the pandemic. SRH records respondent’s evaluation on their health status in COVID-19. SRH is a commonly used measure of personal health status and is a reliable predictor of other health outcomes^37^. Mental health assessment is determined by a revised questionnaire derived from the ten-item version of the Hopkins Symptom Checklist (SCL-10)^38,39^, focusing on respondents’ mental disorder symptoms during the pandemic. The measure is scored such that higher scores reflect increasing levels of psychological mental disorder.

To investigate the intensified discrimination against tenants during the COVID-19 pandemic, we include two dimensions in our analysis: 'expressing discrimination' and 'perceived discrimination.'The first dimension, 'expressing discrimination', measures the extent to which respondents believe that tenants increase the risk of COVID-19 infection in the community. The specific question asked in the questionnaire is, "To what extent do you agree with the statement that renters in the neighborhood will increase the infection risk of COVID-19?" The second dimension, 'perceived discrimination,' measures how tenants perceive discrimination based on their identity as tenants. The question asked is, "To what extent do you agree with the statement that you feel discriminated against because of your identity as a tenant when entering or leaving the neighborhood or other places?" Both questions used a five-point Likert scale to measure the level of discrimination. Echoing Tranter and Donoghue’s classification^4^, four types of housing tenure are included: homeowner without mortgages, homeowner with mortgages, renter living in private housing and renter living in public housing.

Multi-level control variables are included in this analysis: individual level and neighbourhood level. Individual-level control variables include age, gender, employment, income, biomedical and exercise habit. *Hukou* records respondents’ official household registration: rural *hukou* and urban *hukou*, which is also examined as an important dimension of pre-existing identity-based discrimination in China^40^. Neighbourhood-level control variables comprise two categories: physical environment and social capital. The physical environment includes neighbourhood density determined by the architectural height, property management level, recreation facilities and pandemic risk. Following the concept of community social capital^41^, four key aspects are measured in this study: social support, social engagement, social network and social trust. The outcomes are expressed on a five-point scale and we calculate the mean scores of all responses in the same communities to describe the neighbourhood characteristics. More detailed information on measuring the variables in our questionnaire is available in the supplementary file.

For this analysis, we initially received 4880 responses. We then excluded 0.2% (10) records because of the missing values in one of the major factors. We also excluded 9 abnormal records that showed inconsistent answers for same questions. In total, we analysed 4861 survey responses, among which 2628 are tenants. Table 1 shows the variables included in this study.

**Table 1:** Variables description and measurement.

Variables	Variable definition and measurement
Health outcomes	
SRH	Self-rated health: ranging from 1 (dissatisfied) to 5 (very satisfied)
Mental health	Sum of self-reported mental disorder experience;
Discrimination experience	
Expressing discrimination	Linking tenant with infection risk: disagree (1); relatively disagree (2); neutral (3); relatively agree (4); very agree (5);
Perceived discrimination	Perceived discrimination in the community during COVID-19: disagree (1); relatively disagree (2); neutral (3); relatively agree (4); very agree (5);
Housing tenure	Classification: 1 = homeowner without mortgages, 2 = homeowner with mortgages, 3 = tenants living in private housing, 4 = tenants living in public housing;
Individual-level variables	
Age	Dummy variable, 0 = > 60, 1 = 18–25, 2 = 26–35, 3 = 36–60;
Gender	Dummy variable, 0 = male, 1 = female;
Hukou	Dummy variable, 0 = urban hukou, 1 = rural hukou;
Employment	Dummy variable, 0 = employed, 1 = unemployed (including working part-time);
Income	Continuous variable, individual average monthly income;
Biomedical health	Continuous variable, sum of the selected chronic diseases an individual suffers from;
Exercise habit	Continuous variable, frequency of individual monthly outdoor exercise during the pandemic;
Neighborhood-level variables	
Neighborhood density	Dummy variable, determined by the building height: low-rise (1); multi-storey (2); middle-high-rise (3); high-rise (4);
Property management	Continuous variable, number of managing services;
Recreation facilities	Continuous variable, number of public facilities;
Pandemic risk	Continuous variable, rated risk of COVID-19 in the community;
Social support	Continuous variable, measuring to what extent residents could gain necessary help in the community;
Social engagement	Continuous variable, measuring the civic engagement and spontaneous assistance in the community;
Social network	Continuous variable, measuring the social interaction in the community;
Social trust	Continuous variable, measuring to what extent the residents could trust each other in the community;

### Research design

T-test, multi-level regression model and ordered logit model are employed in this study. All the variables are standardised. The t-test is utilised to summarise the health disparities between various housing tenures in different regions. We then perform multi-level regression models to analyse the hierarchical data. The results can reveal the associations between the intensified discrimination and health outcomes during the pandemic, as well as the impact on the associations from various factors at two levels. We specify the model as follows:

1 $$H \, = \, \alpha_{0} + \, \beta_{0} \times \, M \, + \, (\beta_{1} \cdot I_{j} + \, \beta_{2} \cdot N_{k} ) \, + \, \varepsilon ,\,\,\,\,\, \left( {{\text{j }} = { 1},{ 2}, \, \cdots ,{ 8};{\text{ k }} = { 1},{ 2}, \, \cdots ,{ 8}} \right)$$

where *H* is the health outcomes of respondents; *M* represents the tenants’ perception of discrimination; *I*_*j*_ is the individual-level factors; *N*_*k*_ is the neighbourhood-level factors; *β*_*0*_*, β*_*1*_ and β_2_ are the estimated coefficients; and *ɛ* is the error term. A two-level empty model with the intercept and residual is first fitted to examine the random effects, and the intraclass correlation coefficient (ICC) is calculated to determine the efficiency of multilevel model analysis. If ICC = 0, the structure of data is not hierarchical, and the analysis should be simplified to a traditional single-level model. In fitting the two-level model, discrimination factors are first included in the model, and demographic information and health status are added in turn. Neighbourhood-level factors are added thereafter. On the basis of the results, we use the interaction terms to further evaluate the interaction between identity-based discrimination and significant independent variables, and a fully adjusted model is obtained.

**Figure 1:**
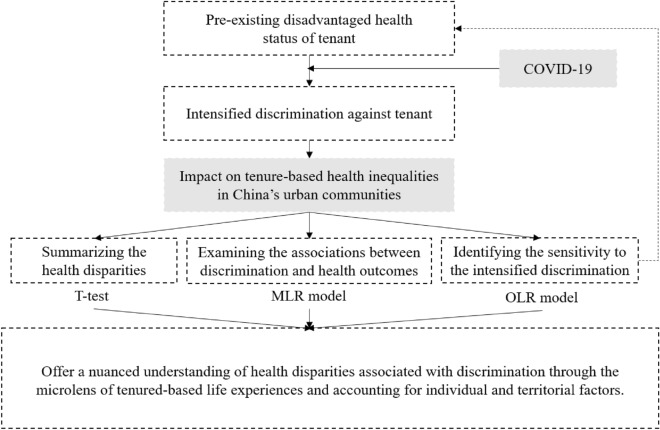
The analytical framework.

Finally, to reveal more nuanced differences of the discriminatory experience, we use the ordered logistic model to examine the social groups sensitive to the intensified discrimination. On the one hand, in light of existing research on the classification of housing tenure, we divide the homeowners into two groups: homeowners with or without mortgages. On the other hand, given the different institutional arrangements of public and private housing in China, we further divide these two housing types. This approach will enable us to explore the unique health implications of each housing type and identify various homeowners’ attitudes to the stigma of renters. Data analysis is performed by StataMP 16, and a *p*-value less than 0.05 is considered statistically significant. Figure 1 shows the analytical framework.

## Results

### Housing tenure and general health disparities

**Figure 2:**
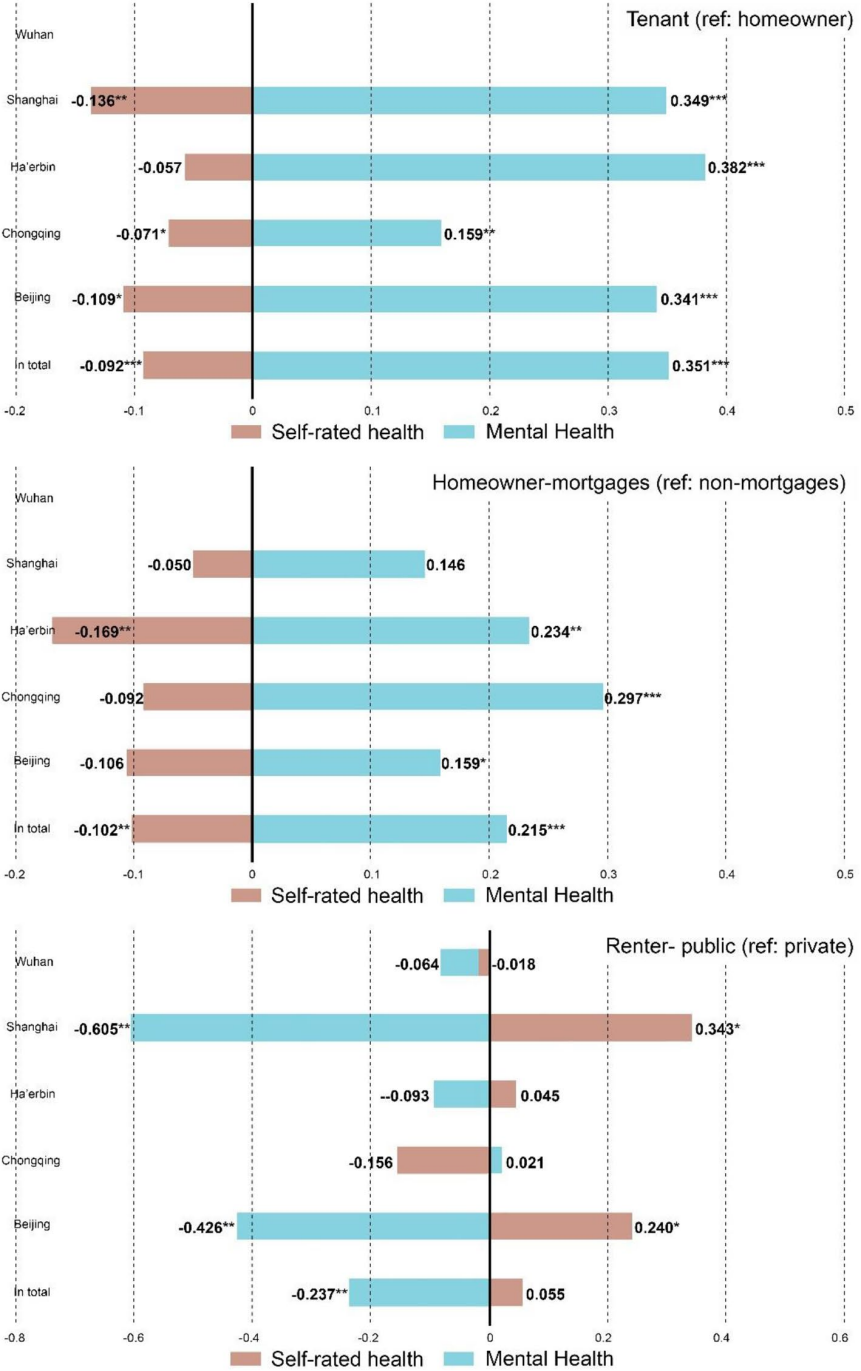
T-test results of discrimination and general health disparities by different groups (the value in brackets refers to the Mean diff. and Sig., respectively). The respondents in Wuhan are all tenants, so the analysis of ‘tenant’ and ‘homeowner-mortgages’ are not applicable in Wuhan.

We first examine the general health disparities for various housing tenures in different cities in China during the COVID-19 pandemic and Fig. 2 shows the results. Approximately 41% of respondents reflect a significant worry about the higher infection risk brought by the tenants living in communities. In general, tenants report lower SRH and higher mental health risks than homeowners. The results also reflect differences between cities, for instance, tenants in Beijing, Chongqing and Shanghai may experience similar SRH gap, while the result for Ha’erbin shows no significant differences. By contrast, the results for mental health are more robust, that is, the tenant identity is associated with higher mental disorder risks in all case cities.

In particular, the *t* test analysis reveals more details on the health disparities among four types of housing tenures. Homeowners with a mortgage are more likely to report worse SRH and mental health than those self-purchased homeowners without mortgages, especially in Beijing, Chongqing and Ha’erbin. By contrast, compared with renters living in private housing, renters living in public housing experience better mental well-being during the pandemic, especially in Beijing and Shanghai. Notably, the tenants living in public housing in Beijing and Shanghai also report higher SRH than those living in private housing, while the results in the other three cities show no significant differences.

### Factors associated with the relationship between discrimination against tenants and health inequalities in COVID-19

The estimation impacts on SRH are shown in Table 2. Model 1 shows the test results of the empty model. The ICC is 0.212, and the level 2 variance of the empty model is statistically significant (*p* < 0.001). Therefore, 21.2% of the total variation is caused by neighbourhood differences, and a multi-level regression model is needed.

**Table 2:** Multi-level models of self-rated health inequalities in COVID-19.

Dependent variable: SRH						
Variables	M 1	M 2	M 3	M 4	M 5	M 6
	Coef. (SE)	Coef. (SE)	Coef. (SE)	Coef. (SE)	Coef. (SE)	Coef. (SE)
Constant	3.848*** (0.012)	3.379*** (0.051)	3.423*** (0.122)	3.577*** (0.152)	3.548*** (0.163)	3.446*** (0.238)
Discrimination factor						
Perceived discrimination		− 0.135*** (0.014)	− 0.129*** (0.014)	− 0.109*** (0.014)	− 0.095*** (0.014)	− 0.111 (0.149)
Interaction term						
Discrimination*housing tenure						0.017 (0.045)
Discrimination*income						− 0.024** (0.007)
Discrimination*exercise habit						0.022* (0.013)
Discrimination*social support						− 0.004* (0.015)
Random variance						
Residual		0.474	0.458	0.448	0.437	0.437
Intercept	0.493	0.146	0.138	0.135	0.128	0.123
-log likelihood	5690.02	2886.84	2837.85	2812.53	2774.00	2767.18

(1) ***, ** and * represent the 0.1%, 1% and 5% significance levels, respectively, (2) the question about discrimination is only asked to tenants. Thus, the variable ‘housing tenure’ in multi-level models comprises only two types: tenant renting private housing and tenant renting public housing. (3) for brevity, the complete table is provided in the supplementary file.

After adjusting all the factors in model 5, tenant’s perceived discrimination remains significantly associated with the SRH in COVID-19. Controlling the individual- and neighbourhood-level factors, the result of ‘perceived discrimination’ is negative and significant at the 0.001 level, indicating that the discriminatory experience increases the SRH gap between homeowners and tenants. Although showing no significant differences, the results of ‘housing tenure’ are positive and consistent in all models, implying that the tenants living in public housing might have a better SRH than tenants living in private housing. The results of community social capital indicate that better social support and social network can enhance residents’ self-rated health outcomes during the pandemic, while the roles of social engagement and social trust are less significant. With respect to other variables, age, personal income, exercise habit, quality of property management and recreation facilities are positively and significantly associated with the SRH outcomes, while unemployment, worse original biomedical health and higher infection risk in the community could bring a negative impact on respondents’ SRH during the pandemic.

After comparing the relevant factors, model 6 is the optimal model revealing the moderating effects of multi-level factors. The result of ‘discrimination*exercise habit’ implies that personal exercise frequency may mitigate the negative health impact of the discrimination against tenants. Similarly, the interactive term of ‘income’ and ‘perceived discrimination’, indicating that a reliable economic situation could narrow the health gap related to discrimination. For neighbourhood-level factors, the result of ‘discrimination*social support’ suggests that the well-organized community resources help alleviate the negative SRH impact of discrimination during the pandemic. This is a clear indication of the mitigation effect of community social capital. Notably, the result of ‘discrimination*housing tenure’ shows no significant differences, indicating that the discriminatory experience might cause a similar negative impact on tenants’ SRH in public housing and private housing.

The estimation impacts on mental health are shown in Table 3. Model 1 shows the test results of the empty model. The ICC is 0.226, and the level 2 variance of the empty model is statistically significant (*p* < 0.001). Therefore, 22.6% of the total variation is caused by neighbourhood differences, and a multi-level regression model should be used.

**Table 3:** Multi-level models of mental health inequalities in COVID-19.

Dependent variable: mental health						
Variables	M 1	M 2	M 3	M 4	M 5	M 6
	Coef. (SE)	Coef. (SE)	Coef. (SE)	Coef. (SE)	Coef. (SE)	Coef. (SE)
Constant	2.663*** (0.018)	3.581*** (0.079)	3.073*** (0.190)	2.468*** (0.234)	1.833*** (0.254)	2.532*** (0.412)
Discrimination factor						
Perceived discrimination		0.225*** (0.022)	0.212*** (0.022)	0.168*** (0.022)	0.162*** (0.022)	0.361 (0.244)
Interaction term						
Discrimination*housing tenure						0.083 (0.070)
Discrimination*hukou						− 0.028* (0.043)
Discrimination*pandemic risk						0.070** (0.024)
Discrimination*property management						− 0.015* (0.014)
Discrimination*social support						0.028* (0.026)
Discrimination*social engagement						0.022* (0.022)
Random variance						
Residual	1.084	1.088	1.071	1.035	1.034	1.024
Intercept	0.317	0.417	0.371	0.347	0.335	0.335
-log likelihood	7665.46	3972.57	3923.29	3872.13	3861.52	3854.29

(1) ***, ** and * represent the 0.1%, 1% and 5% significance levels, respectively, (2) the question about discrimination is only asked to tenants. Thus, the variable ‘housing tenure’ in multi-level models comprises only two types: tenant renting private housing and tenant renting public housing. (3) for brevity, the complete table is provided in the supplementary file.

Model 5 reports that tenant’s perception of discrimination remains significantly associated with mental health in COVID-19. With other factors controlled, the result of ‘perceived discrimination’ is positive and significant on the 0.001 level, suggesting that the discriminatory experience increases the mental health inequalities. Similar to the findings of SRH, the results of ‘housing tenure’ suggest the promoting effect of public housing on tenants’ mental health. Particularly, compared with residents holding urban *hukou*, the result of ‘*hukou’* shows that the residents with rural *hukou* may report more psychological problems. The results of community social capital are mixed, that is, better social support and less social engagement can mitigate mental disorder risks of tenants. This may be attributed to that residents’ active engagement in community affairs during the pandemic could inevitably increase their psychological anxiety. For other demographic and neighbourhood characteristics, the elderly, the female, people with fewer biomedical diseases and less exercise frequency may share better well-being, while the lower neighbourhood density and well-organised property services, lower COVID-19 risk may benefit tenants’ mental health during the pandemic.

After comparing the relevant factors, model 6 is the optimal model reporting the moderating effects of individual and territorial features. The unexpected finding from the analysis of 'discrimination*hukou' suggests that tenants with rural hukou may experience fewer discrimination-related mental health issues than tenants with urban hukou. This could be attributed to the potential moderating effect of the pre-existing discrimination against rural renters in China's household registration system. Similarly, the results of the interactive term of ‘property management’ and ‘perceived discrimination’ suggest that satisfactory property management could mitigate mental health inequalities related to the intensified discrimination. Surprisingly, the result of ‘discrimination*pandemic risk’ shows that a higher COVID-19 risk in the community may reduce the negative mental health impact of discrimination during pandemic. This may be because, as a more direct stressor, higher COVID-19’s infection risk could have overtaken discrimination to become a more significant source of mental health risks. This suggests that COVID-19 may bring more severe psychological distress to residents through a combination of actual infection risks and difficulties in daily life. It is worth noting that the results of community social capital, including ‘discrimination*social engagement’ and ‘discrimination*social support’, indicate that they may reduce the negative effects of discrimination on mental health. These echo the findings in the SRH analysis. Despite of the potential anxiety associated with the participation in community activities, better social capital contributes to narrow the tenure-based mental health inequalities. The result of ‘discrimination*housing tenure’ remains similar to that in SRH’s models.

### Groups sensitive to the intensified discrimination against tenants

Table 4 shows the analysis results of the ‘sensitivity’ to the intensified discrimination within different social groups. Model 1 shows the residents who are more likely to discriminate tenants in COVID-19. That is, these residents argue that the increasing tenants living in their neighbourhoods may significantly increase the infection risk of COVID-19. Although a total of 583 of 2628 tenants also reported that they agree that ‘tenants will increase the infection risk’, however, the results of ‘housing tenure’ indicate that both homeowners without and with mortgages are more likely to express discrimination than tenants during COVID-19. Gender and exercise habit are significantly associated with expressing intensified discrimination, while no significant difference is observed between rural and urban *hukou*. Respondents who are female and exercise less are more likely to express the discrimination on tenants than their counterparts. Compared with the elderly, middle-aged respondents (36–60 years old) have a higher possibility to discriminate against tenants. The result of biomedical health is positive, implying that respondents with more selected chronic diseases discriminate against tenants more during COVID-19.

**Table 4:** Ordered logit regression results.

Variables	Model 1: discriminate		Model 2: vulnerable	
	Coef	Std. err	Coef	Std. err
Age (ref: > 60)				
18–25	0.228	0.159	0.215	0.248
26–35	0.218	0.142	0.014	0.233
36–60	0.298*	0.141	− 0.343	0.238
Female (ref: male)	0.156**	0.054	0.110	0.075
Hukou (ref: urban)	0.109	0.063	0.161*	0.078
Housing tenure	Ref: homeowner without mortgages		Ref: renter in private housing	
Homeowner with mortgages	0.055	0.080	–	–
Renter in private housing	− 0.427***	0.068	–	–
Renter in public housing	− 0.428**	0.132	− 0.354*	0.145
Unemployed (ref: employed)	0.110	0.119	0.119	0.186
Income	− 0.000	0.014	0.089***	0.020
Biomedical health	0.105*	0.050	0.285**	0.087
Exercise habit	− 0.097***	0.025	− 0.103**	0.035
Number of cases	4861		2466	
LR chi2(13)	106.39		71.10	
-Log likelihood	7124.65		3615.20	

(1) ***, ** and * represent the 0.1%, 1% and 5% significance levels, respectively, (2) In model 2, the question about discrimination is only applicable to tenants.

Model 2 reveals the renting groups who are more likely to experience discrimination during the pandemic. Controlling other factors, the results illustrate that the tenants living in public housing perceive less intensified discrimination than those living in private housing. Notably, the result of ‘*hukou*’ implies that the tenants with rural *hukou* perceive more discrimination than tenants with urban hukou. These findings suggest that the migrant identity may further increase the vulnerability of tenants in terms of the intensified discrimination. The analysis results also illustrate that personal income, original biomedical health, and exercise habit are associated with tenants’ perception of discrimination. Respondents with higher exercise frequency are less likely to be affected by the discrimination, whereas the respondents who have high personal income or have poor biomedical health share a higher frequency of perceiving the identity-based discrimination in COVID-19.

## Discussion and conclusions

Studies on housing tenure and health inequalities have long been fertile ground for academic debates and governance innovation. Urban geographers and policymakers have widely and increasingly recognised the health disparities between various housing tenures in contemporary urbanisation^42^. The COVID-19 pandemic provides a unique opportunity to examine the identity-based discrimination and how it influences the (pre-existing) health inequalities among various housing tenures. On the basis of the questionnaire data collected from five major cities in China, this study seeks to examine the intensified discrimination against tenants in COVID-19 and how it has varied and aggravated health inequalities between various housing tenures, with a focus on the mixed impact of individual profiles and territorial characteristics. The results show that the intensified discrimination during COVID-19 brings about a significant negative impact on tenants’ self-rated health and well-being in China; and the spatial heterogeneity plays a critical role in moderating this relationship, while the impacts of physical and social environment are rather mixed. In summary, the contributions of this study are three-fold: (1) It contributes to emergent literature on the consequences of the COVID-19 pandemic on tenants through the lens of identity-based discrimination^18^. (2) This study supplements existing research on the associations between housing tenure and health inequalities by combining individual socio-economic attributes and territorial factors. (3) It provides timely policy implications for more inclusive urban development in the post-pandemic era, especially considering possible future public health emergences.

In general, the pre-existing discrimination against tenants in China has intensified during COVID-19, suggesting the conflicts and hostility between ‘homeowners’ and ‘tenants’. As the COVID-19 risk is considered associated with high mobility and low socio-economic conditions, tenants are deemed unwelcome in China’s urban communities. Homeowners without mortgages and homeowners with mortgages are more likely to discriminate against tenants by connecting infection risk with tenants. Residents with worse original health conditions are more likely to express the intensified discrimination, which might be determined by several individual attributes such as age, gender and exercise habits. The differences between rural and urban *hukou* are insignificant. Echoing findings from other contexts^18^, our research proves that the COVID-19 pandemic has brought significant and negative impacts on the pre-existing adverse position of tenants as a marginal group that may be extended into post-pandemic era.

This study reveals nuanced relationships between housing tenure and health inequalities through the lens of tenants’ discriminatory experiences during the pandemic. Our findings show that the perceived discrimination has led to significant health inequalities in SRH and mental health between tenants and homeowners. Generally, compared with private neighbourhoods, public neighbourhoods could enable a smaller health gap between tenants and homeowners. However, the intensified discrimination seems to have a similar and negative impact on tenants’ health in private housing and public housing. Furthermore, we find varied moderating influences of the individual and territorial factors on discrimination-associated health inequalities. It is found that tenants with worse socio-economic status or worse health conditions suffer more due to the perceived discrimination, while better neighbourhood environments may play a significant mitigation role. Meanwhile, tenants with lower income and worse exercise habits will be exposed to more severe physical health risks, while tenants with rural *hukou* suffer lower health impact from discriminatory experiences than urban *hukou* holders. By contrast, in terms of territorial features, high-quality property services are found to help decrease the health inequalities associated with intensified discrimination. Notably, although engagement in community volunteer work during COVID-19 may lead to higher mental anxiety, echoing the existing evidence^41^, we find that community social capital is generally beneficial to community health and well-being and helps reduce negative health impact of discrimination. According to the theory of social stigma^43^, the perception of discrimination may spread among the tenants and generate a vicious circle, affecting their social interaction and the wider social inclusion process. Nonetheless, community social capital may enable tenants to escape this vicious circle through emphasizing the common interests of neighborhood cohesion, attachment, and sense of belonging^41^. Our study also reports a significant association between the increased pandemic risk in the community and the discrimination-related mental well-being gap, which introduces an important dimension to understand the negative health impact of COVID-19 more holistically. Even in those neighborhoods with low infection risk, COVID-19 could bring significant and negative impact on human well-being in a relatively indirect way. These findings update our understanding of housing tenure, tenants’ perceived discrimination, health inequalities, and their interactions through the lens of the complex intersections between individual experiences in the community and multi-level factors and environments.

Additionally, this study identifies groups that are more vulnerable to intensified discrimination against tenants during the pandemic. In general, discrimination experiences are more likely to be perceived by the tenants who are more disadvantaged before COVID-19, but a particular risk is also identified for those with better socio-economic status. The tenants with poor original health and high income are more likely to feel being discriminated against, whereas the reflections of exercise habits are reversed. Compared with private housing, public housing seems to have a protective effect on renters during COVID-19. A possible explanation is that residents’ similar socioeconomic status and the institutional protection and welfare provision associated with the public rental housing therein may mediate/alleviate the negative impact of the pandemic. *Hukou* is also a significant factor; echoing existing literature, our finding shows that tenants with rural *hukou* are more likely to perceive the nuanced changes in discrimination than urban *hukou* holders. Throughout the recent history after 1950s, the Chinese state has utilized the household registration institution as a means to delineate urban and rural areas, effectively controlling the access of rural migrants to urban resources and opportunities^30,31^. This institutional arrangement has contributed to a significant and long-term exclusion and marginalization of rural migrants in Chinese cities. In this regard, combined with the moderating effect of rural *hukou* showed on the association between discrimination and mental well-being, a possible explanation is that, based on the accumulation mechanism^11^, tenants with rural *hukou* could better adapt and resist to the intensified discrimination because they have been widely discriminated for their rural *hukou* in urban China even before the pandemic^44,45^. Thus, they suffer less from mental well-being loss despite their higher likelihood of perceiving discrimination during COVID-19.

Our findings also provide important policy implications for inclusive and healthy urban development in the post-pandemic era. Given recent concerns surrounding community governance during and after pandemics, our study highlights the need to develop detailed public health policies that can mitigate potential discrimination against tenants based on their mobility status, as well as minimize negative health impacts. Moreover, for China’s housing policy in the post-pandemic era^46,47^, our study confirms that providing more public housing for vulnerable groups might be effective in improving residents’ mental well-being^48^. However, more attention needs to be paid to social relations and physical environments. Governments should be more cautious in planning ‘mixed-tenure communities’ in case of exacerbating discrimination against tenants and increasing tenure-based health inequalities.

## Limitations

Admittedly, several limitations in data collection and research methods have affected the accuracy of our conclusions. First, electronic questionnaires were used in this study to collect data, and citizens who were unable to access the Internet are underrepresented. Second, this study is a cross-sectional survey and does not reflect the causal relationship between the discrimination against tenants and health inequalities during the pandemic. Third, this study will benefit from qualitative studies of citizens’ views and tenants’ perceptions of the intensified discrimination during the COVID-19 pandemic through interviews and field work, especially for the relationship between perceived discrimination and long-term health inequalities, as well as the moderating impact of community social capital on the relationships.

### Supplementary Information

Supplementary Information.

## Data Availability

The datasets generated and analyzed during the current study are not publicly available due to respondents’ private concerns.
