# Supplementary material for: Intensified discrimination against tenants and its health effects during the COVID-19 pandemic in large Chinese cities
Source: Sci Rep. 2023 Dec 15;13:22316. doi: 10.1038/s41598-023-48935-3 (PMC10724241; doi:10.1038/s41598-023-48935-3)
Supplement: Supplementary file 1 — Supplementary Information. [file 41598_2023_48935_MOESM1_ESM.docx]

**Appendix**

**1. The measurement of self-rated health risk during the COVID-19 pandemic in our questionnaire:**

Question: In general, are you satisfied with your health conditions during the pandemic?

A. Very unsatisfied. B. Unsatisfied. C. Neutral. D. Satisfied. E. Very satisfied.

**2. The measurement of mental health risk during the COVID-19 pandemic in our questionnaire:**

Question: Do you have the following experiences during the pandemic period?

(Never=1, occasionally=2, every now and then=3, frequently=4, always=5)

A. I feel lightheaded or dizzy. B. I feel frightened. C. I am scared or worried. D. I feel nervous and anxious. E. I feel guilty. F. I have poor sleeping quality. G. I feel hopeless. H. I feel frustrated. I. I feel lonely. J. I feel that everything is difficult and helpless. K. I feel worthless. L. I feel the pressure from my family/partner. M. I have financial pressure.

**3. The measurement of interviewees’ original biomedical health during the COVID-19 pandemic in our questionnaire:**

Question: Did you suffer from the following conditions before the pandemic?

A. Diabetes. B. Hypertension. C. Hyperlipidemia. D. Asthma. E. Allergy. F. Other disease. G. Nothing.

**4. The measurement of interviewees’ discrimination against renters during the pandemic in our questionnaire:**

Question: To what extent do you agree with the statement that renters in the neighborhood will increase the infection risk of COVID-19?

A. Very agree. B. Agree. C. Neutral. D. Disagree. E. Very disagree.

**5. The measurement of interviewees’ perception of discrimination during the pandemic in our questionnaire:**

Question: To what extent do you agree with the statement that you feel being discriminated against when entering or leaving the neighborhood or other places because of your identity as a tenant?

A. Very agree. B. Agree. C. Neutral. D. Disagree. E. Very disagree.

**6. The four aspects of community-level social capital during the pandemic are determined by the following questions:** (1) do you think this community can meet your needs? (2) how often do you participate in the public affairs within the community? (3) do you think you have good social relations with other residents of the community? (4) do you think you can trust people in the community?

**7. Supplementary Table 2. Multi-level models of self-rated health inequalities in COVID-19.**

| Dependent variable: SRH | | | | | | |
| --- | --- | --- | --- | --- | --- | --- |
| Variables | M 1 | M 2 | M 3 | M 4 | M 5 | M 6 |
|  | Coef. (SE) | Coef. (SE) | Coef. (SE) | Coef. (SE) | Coef. (SE) | Coef. (SE) |
| Constant | 3.848^***^ (0.012) | 3.379^***^ (0.051) | 3.423^***^ (0.122) | 3.577^***^ (0.152) | 3.548^***^ (0.163) | 3.446^***^ (0.238) |
| **Discrimination factor** | | | | | | |
| Perceived discrimination |  | -0.135^***^ (0.014) | -0.129^***^ (0.014) | -0.109^***^ (0.014) | -0.095^***^ (0.014) | -0.111 (0.149) |
| **Individual-level** | | | | | | |
| Age (ref: > 60) | | | | | | |
| 18-25 |  |  | -0.293^**^ (0.108) | -0.285^**^ (0.107) | -0.232^*^ (0.105) | -0.222^*^ (0.105) |
| 26-35 |  |  | -0.233^*^ (0.101) | -0.231^*^ (0.100) | -0.181 (0.099) | -0.170 (0.099) |
| 36-60 |  |  | -0.270^**^ (0.104) | -0.266^*^ (0.103) | -0.228^*^ (0.102) | -0.228^*^ (0.101) |
| Female (ref: Male) |  |  | -0.024 (0.032) | -0.025 (0.032) | -0.037 (0.032) | -0.040 (0.032) |
| Hukou (ref: urban) |  |  | -0.051 (0.033) | -0.051 (0.033) | -0.057 (0.032) | -0.061 (0.032) |
| Housing tenure (ref: rent-private) |  |  | 0.058 (0.059) | 0.060 (0.058) | 0.048 (0.057) | 0.003 (0.127) |
| Unemployed (ref: Employed) |  |  | -0.199^*^ (0.081) | -0.196^*^ (0.080) | -0.209^**^ (0.079) | -0.209^**^ (0.079) |
| Income |  |  | 0.019^*^ (0.009) | 0.019^*^ (0.009) | 0.016 (0.009) | 0.082^***^ (0.022) |
| Biomedical health |  |  | -0.215^***^ (0.035) | -0.203^***^ (0.035) | -0.178^***^ (0.035) | -0.180^***^ (0.035) |
| Exercise habit |  |  | 0.098^***^ (0.015) | 0.098^***^ (0.015) | 0.079^***^ (0.015) | 0.020 (0.037) |
| **Neighborhood Characteristics** | | | | | | |
| Neighborhood density (ref: Low-rise) | | | | | | |
| Multi-storey |  |  |  | 0.008 (0.081) | -0.037 (0.080) | -0.030 (0.080) |
| Middle-high-rise |  |  |  | 0.024 (0.081) | -0.044 (0.080) | -0.037 (0.080) |
| High-rise |  |  |  | -0.029 (0.080) | -0.066 (0.079) | -0.062 (0.079) |
| Property management |  |  |  | 0.026^*^ (0.012) | 0.017 (0.012) | 0.017 (0.012) |
| Recreation facilities |  |  |  | 0.033^*^ (0.016) | 0.016 (0.016) | 0.017 (0.016) |
| Pandemic risk |  |  |  | -0.120^***^ (0.019) | -0.105^***^ (0.019) | -0.109^***^ (0.019) |
| Social support |  |  |  |  | 0.063^**^ (0.024) | 0.074 (0.048) |
| Social engagement |  |  |  |  | 0.024 (0.019) | 0.024 (0.019) |
| Social network |  |  |  |  | 0.073^**^ (0.023) | 0.071^**^ (0.023) |
| Social trust |  |  |  |  | 0.039 (0.024) | 0.039 (0.024) |
| **Interaction term** | | | | | | |
| Discrimination*Housing tenure |  |  |  |  |  | 0.017 (0.045) |
| Discrimination*Income |  |  |  |  |  | -0.024^**^ (0.007) |
| Discrimination*Exercise habit |  |  |  |  |  | 0.022^*^ (0.013) |
| Discrimination*Social support |  |  |  |  |  | -0.004^*^ (0.015) |
| **Random variance** | | | | | | |
| Residual | 0.493 | 0.474 | 0.458 | 0.448 | 0.437 | 0.437 |
| Intercept | 0.126 | 0.146 | 0.138 | 0.135 | 0.128 | 0.123 |
| -log likelihood | 5690.02 | 2886.84 | 2837.85 | 2812.53 | 2774.00 | 2767.18 |

Note: (1) ***, ** and * represent the 0.1%, 1% and 5% significance levels, respectively; (2) the question about discrimination is only asked to tenants. Thus, the variable ‘housing tenure’ in multi-level models comprises only two types: tenant renting private housing and tenant renting public housing.

**8. Supplementary Table 3. Multi-level models of mental health inequalities in COVID-19.**

| Dependent variable: Mental health | | | | | | |
| --- | --- | --- | --- | --- | --- | --- |
| Variables | M 1 | M 2 | M 3 | M 4 | M 5 | M 6 |
|  | Coef. (SE) | Coef. (SE) | Coef. (SE) | Coef. (SE) | Coef. (SE) | Coef. (SE) |
| Constant | 2.663^***^ (0.018) | 3.581^***^ (0.079) | 3.073^***^ (0.190) | 2.468^***^ (0.234) | 1.833^***^ (0.254) | 2.532^***^ (0.412) |
| **Discrimination factor** | | | | | | |
| Perceived discrimination |  | 0.225^***^ (0.022) | 0.212^***^ (0.022) | 0.168^***^ (0.022) | 0.162^***^ (0.022) | 0.361 (0.244) |
| **Individual-level** | | | | | | |
| Age (ref: > 60) | | | | | | |
| 18-25 |  |  | 0.373^*^ (0.167) | 0.323^*^ (0.164) | 0.320 (0.164) | 0.337^*^ (0.164) |
| 26-35 |  |  | 0.350^*^ (0.157) | 0.319^*^ (0.154) | 0.315^*^ (0.154) | 0.331^*^ (0.154) |
| 36-60 |  |  | 0.229 (0.161) | 0.183 (0.158) | 0.187 (0.158) | 0.207 (0.158) |
| Female (ref: Male) |  |  | -0.149^**^ (0.050) | -0.145^**^ (0.050) | -0.136^**^ (0.049) | -0.131^**^ (0.049) |
| Hukou (ref: urban) |  |  | 0.275^***^ (0.051) | 0.271^***^ (0.050) | 0.274^***^ (0.050) | 0.351^**^ (0.126) |
| Housing tenure (ref: rent-private) |  |  | -0.168 (0.091) | -0.170 (0.089) | -0.164 (0.089) | -0.372 (0.197) |
| Unemployed (ref: Employed) |  |  | -0.170 (0.125) | -0.166 (0.123) | -0.166 (0.122) | -0.156 (0.122) |
| Income |  |  | -0.004 (0.014) | -0.003 (0.014) | -0.002 (0.014) | -0.000 (0.014) |
| Biomedical health |  |  | 0.360^***^ (0.055) | 0.332^***^ (0.054) | 0.319^***^ (0.054) | 0.305^***^ (0.054) |
| Exercise habit |  |  | 0.043^*^ (0.024) | 0.037 (0.023) | 0.048^*^ (0.024) | 0.048^*^ (0.024) |
| **Neighborhood Characteristics** | | | | | | |
| Neighborhood density (ref: Low-rise) | | | | | | |
| Multi-storey |  |  |  | 0.314^*^ (0.125) | 0.338^**^ (0.124) | 0.356^**^ (0.124) |
| Middle-high-rise |  |  |  | 0.312^*^ (0.124) | 0.330^**^ (0.124) | 0.350^**^ (0.124) |
| High-rise |  |  |  | 0.227 (0.123) | 0.238 (0.122) | 0.260^*^ (0.123) |
| Property management |  |  |  | -0.059^**^ (0.018) | -0.057^**^ (0.018) | -0.015 (0.043) |
| Recreation facilities |  |  |  | -0.041 (0.025) | -0.031 (0.025) | -0.030 (0.025) |
| Pandemic risk |  |  |  | 0.259^***^ (0.030) | 0.245^***^ (0.030) | 0.056 (0.070) |
| Social support |  |  |  |  | -0.125^**^ (0.037) | -0.204^*^ (0.083) |
| Social engagement |  |  |  |  | 0.073^*^ (0.030) | 0.010 (0.068) |
| Social network |  |  |  |  | -0.032 (0.037) | -0.028 (0.036) |
| Social trust |  |  |  |  | -0.019 (0.037) | -0.020 (0.037) |
| **Interaction term** | | | | | | |
| Discrimination*Housing tenure |  |  |  |  |  | 0.083 (0.070) |
| Discrimination*Hukou |  |  |  |  |  | -0.028^*^ (0.043) |
| Discrimination* Pandemic risk |  |  |  |  |  | 0.070^**^ (0.024) |
| Discrimination* Property management |  |  |  |  |  | -0.015^*^ (0.014) |
| Discrimination* Social support |  |  |  |  |  | 0.028^*^ (0.026) |
| Discrimination* Social engagement |  |  |  |  |  | 0.022^*^ (0.022) |
| **Random variance** | | | | | | |
| Residual | 1.084 | 1.088 | 1.071 | 1.035 | 1.034 | 1.024 |
| Intercept | 0.317 | 0.417 | 0.371 | 0.347 | 0.335 | 0.335 |
| -log likelihood | 7665.46 | 3972.57 | 3923.29 | 3872.13 | 3861.52 | 3854.29 |

Note: (1) ***, ** and * represent the 0.1 %, 1 % and 5 % significance levels, respectively; (2) the question about discrimination is only asked to tenants. Thus, the variable ‘housing tenure’ in multi-level models comprises only two types: tenant renting private housing and tenant renting public housing.
